# Fluorescence of coral larvae predicts their settlement response to crustose coralline algae and reflects stress

**DOI:** 10.1098/rspb.2010.2344

**Published:** 2011-01-26

**Authors:** C. D. Kenkel, M. R. Traylor, J. Wiedenmann, A. Salih, M. V. Matz

**Affiliations:** 1 Integrative Biology Section, University of Texas at Austin, Austin, TX, USA; 2 National Oceanography Centre, University of Southampton, Southampton, UK; 3 School of Natural Sciences, University of Western Sydney, Penrith, New South Wales 1797, Australia

**Keywords:** genetics, recruitment, dispersal, heritability, GFP, metamorphosis

## Abstract

Multi-coloured homologues of the green fluorescent protein generate some of the most striking visual phenomena in the ocean. Despite their natural prominence in reef-building corals and widespread use in biotechnology, their biological role remains obscure. Here, we experimented with larvae of *Acropora millepora* to determine what can be learned about a coral larva or recruit from its fluorescent colour. We performed 12 crosses between seven *A. millepora* colonies representing differing fluorescence phenotypes, the larvae of which were exposed to a natural settlement cue (crustose coralline algae) and heat–light stress. Parental effects explained 18 per cent of variation in colour and 47 per cent of variation in settlement. The colour of the larval family emerged as a predictor of the settlement success: redder families were significantly less responsive to the provided settlement cue (*p* = 0.006). This relationship was owing to a correlation between parental effects on settlement and colour (*r*^2^ = 0.587, *p* = 0.045). We also observed pronounced (16%) decline in settlement rate, as well as subtle (2%), but a statistically significant decrease in red fluorescence, as a consequence of heat–light stress exposure. Variation in settlement propensity in *A. millepora* is largely owing to additive genetic effects, and is thought to reflect variation in dispersal potential. Our results suggest an optical signature to discriminate between long- and short-range dispersing genotypes, as well as to evaluate stress. Further research in this direction may lead to the development of field applications to trace changes in coral life history and physiology caused by global warming.

## 1. Introduction

Corals are the engineers, builders and bricks of the most biologically diverse ecosystem in the ocean. The future of coral reefs depends on the ability of corals to track the ongoing climate change with acclimatization or adaptation [1,2]. Multi-coloured green fluorescent protein (GFP)-like fluorescent proteins (FPs), which attain their greatest diversity in reef-building corals [3], represent a highly visible (literally) indication that our understanding of these issues is far from complete. Previous work has shown that FPs can be among the most abundant proteins in a coral [4], are strongly upregulated in response to light [5], downregulated by heat stress [6–8] and might be upregulated by injury [9,10]. The extant colour diversity of coral FPs evolved under positive selection, indicating functional importance [11]. All these observations indicate that coral FPs are part of some major mechanism by which a coral interacts with its environment; however, the details of this interaction remain unclear [12,13].

The range of proposed FP functions is remarkably broad and includes modulation of photosynthesis [14–16] or other aspects of physiology [11,17] of the intracellular algal symbionts (‘zooxanthellae’), aposematic colouring or masking the presence of algal pigments from fish herbivores [18], attraction of free-living zooxanthellae [19], sensory function mediated by light-driven electron transport [20], and oxidative stress response through either superoxide quenching [21] or hydrogen peroxide scavenging [22]. While there is broad consensus on the importance of FPs in coral ecophysiology, the complexity of adult coral ecology and the possibility of unaccountable environmental influences on FP expression render testing these hypotheses difficult.

Coral larvae and recruits also display fluorescence, which has been used to develop field survey methods [23]. Coral larvae in most non-brooding species are aposymbiotic [24], numerous and easily reared in a laboratory, providing a convenient model to study functional and genetic aspects of coral fluorescence in a fully controlled setting [25]. We explored genetic, physiological and life-history correlates of larval fluorescence in the reef-building coral, *Acropora millepora*, using a series of full-sibling larval families obtained by cross-fertilizing diversely coloured adult corals during a yearly mass-spawning event.

## 2. Material and methods

### (a) Larval rearing

**Figure 1.**
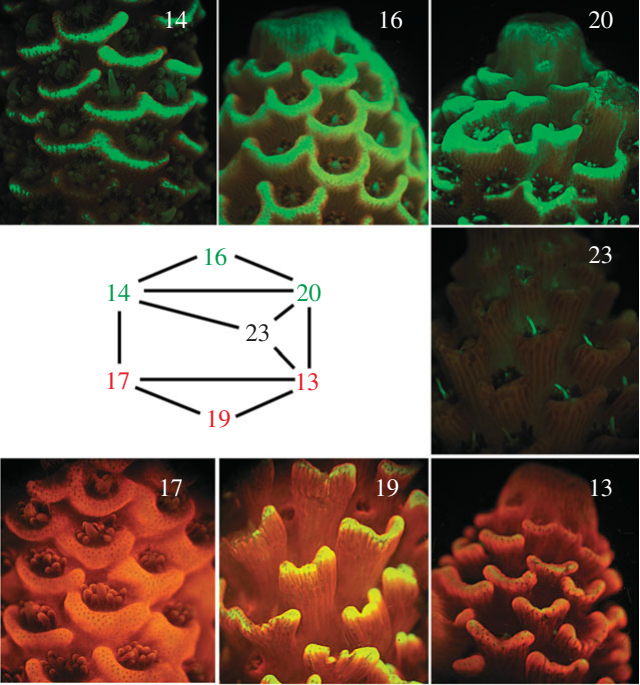
Fluorescent phenotypes of the seven parental *A. millepora* colonies and the crossing design.

Twelve full-sibling families of larvae were obtained by crossing individual parent colonies of *A. millepora* during the November 2006 spawning at Geoffrey Bay, Magnetic Island, Queensland, Australia (19°9′16″ S, 146°51′45″ E). The parental colonies were selected to represent a range of fluorescent phenotypes, including three bright green morphs, three bright red morphs and one non-fluorescent morph (with the exception of the bright green enlarged ‘feeding tentacle’, one per polyp; figure 1). The crosses were performed by combining approximately 500 gamete bundles from each parent in 100 ml of 1.0 µm filtered sea water (FSW) + 50 µg ml^−1^ Ampicillin. The first water change was performed 1 h after fertilization to wash off excess sperm. Water changes were performed twice daily over the subsequent 3 days, after which water changes were performed daily for the duration of the experiment. Ambient temperature was kept between 27°C and 29°C for the initial 5 days post-fertilization that preceded settlement experiments. Four days after fertilization, fluorescence photographs were taken of approximately 18–25 actively swimming larvae haphazardly selected from within each family.

### (b) Settlement induction and stress treatment

On the morning of the fifth day, larvae from each family were split into batches of 50–70 in 15 cm Petri dishes filled with FSW (four plates per family). All plates were exposed to a natural settlement cue, ground-up crustose coralline algae (CCA) [26], representing several unidentified species collected locally. The ground-up CCA were ‘aged’ by keeping them overnight at room temperature in a small volume of sea water, followed by rinsing them twice with sea water immediately before application. This procedure sufficiently weakens the cue for between-family variation in settlement response to be revealed [25]. The four plates comprising each family were then split into two treatments. Two plates were subject to mild stress conditions, consisting of 30 ± 1°C and higher light (200 ± 20 µmol cm^−2^) during the day. The remaining two plates for each family were kept at 28 ± 1°C and in dim light (20 ± 5 µmol cm^−2^) during the day. Light was provided by metal-halide lamps. At night, all plates were kept in the dark at 27 ± 1°C. After 3 days of this treatment, fluorescence photographs were taken of the larvae that remained swimming and of the recruits that settled in a position allowing for photography. All the recruits and remaining larvae were counted to determine the proportion of the family that responded to a settlement cue.

### (c) Fluorescence imaging and quantification

Adult fluorescence was imaged using a fluorescent stereomicroscope MZ FL-III (Leica, Bannockburn, IL, USA) equipped with a Y-GFP-LP long-pass filter (Chroma no. 41029) and a Canon G6 camera. Larval fluorescence was imaged using the double-bandpass F/R filter (Chroma no. 51004v2). To document fluorescence, larvae were immobilized by placing them in 0.04 per cent paraformaldehyde in FSW and photographed immediately. Photos were processed using the photo analysis program ImageJ (W. Rashband, NIMH, Bethesda, MD, USA). Larvae that were not oriented in a lateral view were excluded from analysis as oral or aboral views result in skewed colour values. For all larvae that were positioned in the lateral view, a circle of fixed size, sufficient to encompass the entire larva, was positioned over each individual larva. Red and green raw integrated density colour values were recorded for the area of the circle. A blank of dark background was also measured for each photograph to control for differences in background intensity between photographs. The blank values were subtracted from each larval reading.

### (d) Statistical analysis

**Figure 2.**
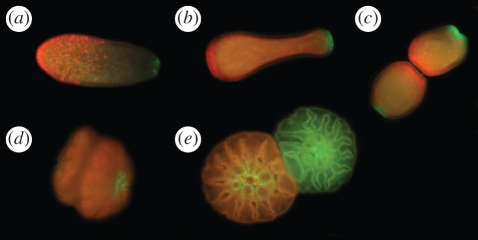
Fluorescence of *A. millepora* larvae and recruits. (*a*) Five-day old larva competent for settlement. Note the green fluorescence in the oral pole (pointing right) and scattered bright red fluorescent ectodermal cells in the aboral pole. (*b*) Larva after exposure to a settlement cue (crustose coralline algae). Note the characteristic ‘bowling pin’ shape and strong aggregation of red fluorescence on the aboral pole. (*c*) Onset of metamorphosis. (*d*) Advanced metamorphosis. On (*c*,*d*), two larvae settle on each other rather than on the settlement substrate, which allows for a better view of the process. (*e*) Two full-sibling recruits one day post-metamorphosis. Note developing mesenteries and tentacles, and persisting green fluorescence at the mouth.

Because the absolute intensity of our photographs could vary, we based the colour analysis on a relative colour measure: the ‘redness’ of individual larvae, calculated as a proportion of red in the total (red + green) colour intensity. For settlement analysis, we used the proportion of individuals in the family that responded to the settlement cue by settlement and metamorphosis (figure 2*e*) over the course of 3 days. Colour and settlement values were arcsin square root-transformed prior to the analysis, as is standard practice for percentage data [27]. All the statistical procedures were performed using R software [28]. The parental effects on colour and settlement were investigated in a series of multiple regression models, involving presence or absence of each parent from a particular cross as factors. Other comparisons were performed in a series of linear mixed models using the *lme4* package [29]. The effect of stress on settlement was modelled with treatment (control or stress) as a fixed factor and replicate nested within family as random factors. To investigate the colour changes, two types of comparison were performed. First, we asked whether the colour of the progeny changed after the settlement induction when compared with the colour of uninduced larvae. This model incorporated the time of colour measurement (pre- or post-induction) as a fixed factor and random effects of replicate nested within treatment nested within family. In the second comparison, we asked whether the heat–light treatment influenced the colour of either recruits or remaining swimming larvae, or both. In the full model (M2), treatment and status (recruit or larva) were included as fixed factors, as well as the interaction term. In another model (M1), the only fixed factor was treatment. The random effects for both these models were replicates nested within family. For all linear mixed models, the random effects were modelled as scalars, as we found that inclusion of random slopes does not improve the fit of the models significantly (likelihood ratio test, *p* ≥ 0.16). The nominal *p*-values for the significance of fixed factors were derived via Monte Carlo Markov chain (MCMC) simulations using the functions *mcmcsamp* and *HPDinterval* of the *lme4* package.

## 3. Results

### (a) Fluorescence patterns in larvae and recruits

Fluorescence of larvae and recruits was consistent with previous observations of the same species [30]. Four-day old larvae displayed green fluorescence in the epidermis surrounding the larval mouth, scattered bright red epidermal cells predominantly on the aboral pole and weaker fluorescence of the gastrodermis ranging in colour from green to red (figure 2*a*). The exposure to a natural settlement cue (CCA) prompted larvae to assume a characteristic ‘bowling pin’ morphology, with the red epidermal cells concentrated strongly on the aboral pole (figure 2*b*). During metamorphosis (figure 2*c*,*d*) the red epidermal cells seem to disappear, while green fluorescence around the mouth and gastrodermal fluorescence persist into the recruit stage (figure 2*e*).

### (b) Parental effects on colour and settlement

**Figure 3.**
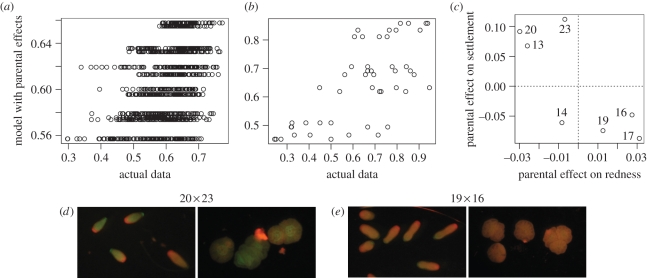
Parental effects. (*a*) Redness: correlation between the predictions of multiple regression model with parental effects and the actual data, *r*^2^ = 0.182, *p* < 10^−15^. (*b*) Same as (*a*), for settlement, *r*^2^ = 0.472, *p* = 0.00012. (*c*) Correlation between parental effects on redness and settlement, *r*^2^ = 0.587, *p* = 0.045. The labels at the points identify parental colonies. On panels (*a*–*c*), the points are plotted on the data scale, while the correlation measures are reported for arcsine square root-transformed values. (*d*) Post-induction fluorescence of larvae and recruits for two of the most contrasting full-sibling families. The numbers on top denote parental colonies.

Redness values of larvae varied significantly across families (figure 3*d*; *F*_11,308_ = 5.63, *p* < 10^−4^). Larval families also exhibited large and statistically significant differences in their response to the provided settlement cue, ranging from 37 to 94 per cent settlement under non-stressful conditions (*F*_11,12_ = 3.19, *p* = 0.029). Linear models suggested that in our experiment, parental effects were the strongest predictors of both colour and settlement, explaining 18 per cent of variation in colour and 47 per cent of variation in settlement (figure 3*a*,*b*; table 1; complete analysis of variance (ANOVA) and model results are available in electronic supplementary material). Moreover, parental effects on colour and settlement were correlated (ANOVA: *F*_1,5_ = 7.11, *p* = 0.045; Pearson's rank correlation: *p* = 0.048; figure 3*c*). However, there was no detectable correlation between the colour of the adult and colour of the offspring: for example, the red colony 13 tended to produce some of the greenest progeny, whereas the green colony 16 produced red offspring (figures 1 and 3*c*).

**Table 1.** Results of multiple regression models exploring parental effects.

response	modelled factors	*r*^2^	*p*
redness	stress treatment + recruitment status	0.0826	<2.2 × 10^−16^
	parental effects	0.182	<2.2 × 10^−16^
	combined	0.2505	<2.2 × 10^−16^
settlement	stress treatment	0.1515	0.0063
	parental effects	0.4718	0.00012
	combined	0.6233	7.25 × 10^−7^

### (c) Colour change from pre- to post-induction of settlement

**Figure 4.**
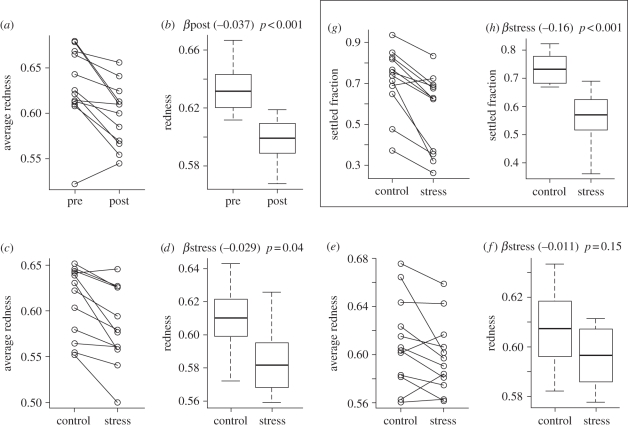
Effect of various conditions on fluorescent colour (redness) and settlement. (*a*,*b*) Change in colour between two time points of measurement, pre- and post-exposure to the settlement cue. (*c*,*d*) Effect of heat–light stress on colour of recruits. (*e*,*f*) Effect of heat–light stress on colour of the larvae that failed to metamorphose. (*g*,*h*) Effect of heat–light stress on the proportion of settled individuals. In each pair of graphs, the left panel shows raw data (points represent family-specific averages, lines connect points for the same family across treatments), and the right panel shows the results of the linear mixed model (boxes represent estimated means ± s.e., conditional on random effects of families; whiskers denote quartiles of the raw data; the numbers on top give the slope of the reaction norm *β* and its significance).

The difference between the colour of the families pre- and post-induction was subtle (average decrease in redness by 3.7%) but strongly significant (*p*_MCMC_ < 10^−4^; figure 4*a*,*b*). Nearly identical colour change was detected when the post-induction data included only recruits or only larvae, indicating that recruits and larvae were affected similarly.

### (d) Effect of stress

According to model M1, which did not partition the colour variance into recruits and larvae, stress resulted in a 2.1 per cent decrease in redness, which was strongly significant (*p*_MCMC_ < 10^−4^). Model M2 revealed that stress mostly affected recruits, resulting in a 2.9 per cent redness decrease (*p*_MCMC_ = 0.04; figure 4*c*,*d*), whereas in larvae the effect was less (1.1% redness decrease) and was not statistically significant (*p*_MCMC_ = 0.15; figure 4*e*,*f*). Stress affected the larval response to a settlement cue much more strongly than colour, resulting in average 16 per cent decrease in settlement (*p*_MCMC_ < 10^−4^; figure 4*g*,*h*).

### (e) Correlation between colour and settlement

**Figure 5.**
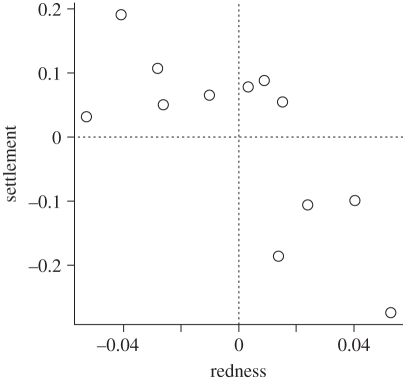
Correlation between modelled family-specific effects on settlement and redness. The points are plotted on the data scale, while the correlation measures are reported for arcsine square root-transformed values. *r*^2^ = 0.562, *p* = 0.005.

The family-specific effects on colour and settlement computed by our linear mixed models were strongly correlated (ANOVA: *F*_1,10_ = 12.05, *p* = 0.005; Pearson's rank correlation: *p* = 0.006; figure 5). The family-specific effects inferred by the models describe how a particular family performed overall across all treatments, either with respect to settlement or colour. In our datasets, these values were nearly identical to the family-specific averages of colour or settlement across all treatments and replicates (*r*^2^ > 0.99 in both cases). It is important to note that these values come from completely independent datasets, one for colour and another for settlement.

## 4. Discussion

### (a) Parental effects

Parental effects were responsible for most of the colour and settlement variation explained by our models (table 1). In our analysis, they represent additive effects owing to genetics, plus potential contribution of non-genetic maternal and paternal factors. The proportion of variation in settlement explained by parental effects (47%) matches remarkably well with our previous estimate of the additive genetic component of settlement variation in the same species, *h*^2^ = 0.49 [25], indicating that at least for this trait, genetics is responsible for most if not all of the parental effect. This result confirms that responsiveness to settlement cues in natural populations of *A. millepora* varies greatly owing to additive genetic effects. The magnitude of this variation (figure 4*g*), as well as the frequency of low-responsive genotypes (four out of seven in the current experiment, figure 3*c*, and two out of three in the experiment described earlier [25]), suggests that this variation may be adaptive, possibly maintained by balancing selection. Delayed or generally weaker response to settlement cues is thought to reflect the tendency for long-range dispersal [25,31]. Genotypes that settle quickly would be better at reseeding the same reef, a good strategy in a stable environment. In turn, genotypes dispersing over longer distances would be favoured under changing environmental conditions, enabling the corals to re-colonize heavily degraded reefs or to achieve a latitudinal range shift in response to global warming [32,33]. The correlation between the magnitude of response to a settlement cue and potential dispersal range, as well as the presence of balancing selection for this trait, can be verified in the future by analysing population genetics of alleles at the associated quantitative trait loci (QTL).

The lack of relationship between the colour of the parent (figure 1) and the parent's effect on colour of the progeny (figure 3*c*) could have been expected, as analysis of the sequence data obtained from *A. millepora* adults [3] and larvae [30] indicates that larvae and adults express different sets of genes coding for GFP-like proteins (M. Matz 2010, unpublished data). The most unexpected result of the present study was the correlation between parental effects on colour and settlement (figure 3*c*): parents producing redder larval offspring also conferred weaker settlement propensity to the progeny. Although the correlation is formally significant (*p* = 0.045), this result must be treated with caution considering the small sample size (*n* = 7 parental genotypes), and would benefit from validation in the future. The nature of the parental effect on colour also merits further inquiry, as our experimental design did not allow for disentangling of additive genetic variation from non-genetic parental effects (such as, for example, yolk content of the eggs).

### (b) Colour changes

In summary, the larvae became greener between the two points of colour measurement (pre- and post-induction; figure 4*a*), which was more pronounced under mild heat–light stress, especially in recruits (figure 4*c*,*e*). Beltran-Ramirez [30] previously reported moderate downregulation of transcription of GFP-like proteins in larvae of *A. millepora* undergoing settlement and metamorphosis, although he could not discriminate between green and red FP transcripts. This result suggests that the colour change in our experiments is probably owing to differential down- rather than upregulation of genes coding for red or green FPs. Still, this is unexpected, as the half-life of FPs in adult corals was reported to be in the order of 20 days [34], which would effectively prevent any transcriptional downregulation from having an effect within the timescale of our experiment. It is possible that turnover of FPs in coral larvae is faster than in adults, or that specific mechanisms are responsible for their relatively rapid disappearance. The fact that the greening was similar in larvae and recruits indicates that it is unlikely to be related to metamorphosis *per se*, but may be a reaction to the settlement cue (even though some individuals failed to respond to it), or it may simply be a function of time. As we did not quantify mortality in our experiment, we also cannot rule out the possibility of preferential mortality of redder individuals.

The amplifying effect of heat–light stress (figure 4*c*,*e*) could also be attributed to the same time-dependent greening, as coral larvae are known to develop faster at a higher temperature [35,36]. However, the effect of stress in recruits was more pronounced than in larvae, suggesting involvement of a specific response on top of the possible increase in metabolic rate. Heat has been shown to downregulate FP expression in *A. millepora* larvae [6–8], whereas light differentially upregulated various colour types of FPs in *A. millepora* [5]. Specifically, green FP was upregulated in the tentacles of *A. millepora* recruits in response to blue light [5]. As we observed recruits becoming greener upon heat–light exposure (figure 4*c*) in our experiment, the inducing effect of higher light had to be more pronounced than potentially inhibiting effect of heat. Our results add to the series of arguments presented earlier [5,11,12] that the function of coral FPs is not necessarily linked to coral–zooxanthellae symbiosis, as fluorescence responds to environmental factors in larvae and recruits that lack symbiotic algae.

### (c) Effect of stress on settlement

We observed strong decline in settlement rate under heat–light stress conditions, amounting to 16 per cent on average across larval families (figure 4*d*). This result is in agreement with several earlier reports for other corals [36–38], and suggests that corals may fail to maintain settlement rates as global warming progresses. However, there have been reports to the contrary. Nozawa & Harrison [39] observed elevated settlement rate in *Acropora solitaryensis* and *Favites chinensis* at higher temperatures, although the post-settlement mortality was also greater. A recent study based on multi-year field data from Japan [40] reported that coral recruitment was unaffected during the thermal anomaly of 1998. It is possible that the effect of stress on settlement may vary depending on experimental conditions. In our experiment, for example, the decline of settlement may be owing to the effect of elevated light rather than temperature, as larvae become negatively phototactic during the settlement phase [37,41,42]. In the future, more studies based on field observations may clarify this issue.

### (d) Colour-settlement correlation

Regardless of the exact nature of parental effect on colour, or the significance of its correlation with the parental effect on settlement, the average colour of the larvae and recruits emerges as an indicator of the settlement propensity of the particular family, explaining 56.2 per cent of settlement variation (figure 5). This correlation may be owing to possible involvement of the coral red FP in sensory functions, which has been hypothesized previously [20,30]. Alternatively, coral FPs may be involved in some function that is not part of the settlement process *per se*, but accompanies it, such as calcification [5]. Finally, taking into account the tentative correlation between parental effects (figure 3*c*), it is possible that colour and settlement covary not because of any functional relationship, but simply because major QTLs for colour and settlement are closely positioned in the coral genome. Interestingly, on the physiological rather than genetic level, the decrease in redness does not necessarily indicate higher settlement potential: in the heat–light stress experiment presented here, the larvae became greener but also less likely to settle (figure 4*c*–*h*).

Our results suggest the possibility of using the colour of recruits as an optical signature to evaluate the distribution and settlement success of low- and high-responsive genotypes *in situ*. This would facilitate the study of factors affecting genetic variation in settlement propensity, and in particular, of testing the hypothesis of correlation between settlement propensity and dispersal range. Before our result can be applied in such studies, however, it is necessary to test the extent to which the colour–settlement correlation holds when analysing individual larvae rather than whole families; in the field as well as in the laboratory. It can be envisioned that if the colour–settlement correlation stands up to such scrutiny, it could lead to practical applications in reef monitoring, for the first time providing coral reef managers with a tool to assess the ecological patterns and adaptive trends of such a fundamental parameter of coral life history as dispersal range.
